# Epidemic Cholera

**Published:** 1848-01

**Authors:** 


### EPIDEMIC CHOLERA.

This fearful epidemic appears to be advancing upon Europe in a course very similar to the one it pursued sixteen years ago. We have not had leisure to prepare our promised notice of its progress for this number, but hope we shall be able to do so for our next.

